# An atlas of spider development at single-cell resolution provides new insights into arthropod embryogenesis

**DOI:** 10.1186/s13227-024-00224-4

**Published:** 2024-05-10

**Authors:** Daniel J. Leite, Anna Schönauer, Grace Blakeley, Amber Harper, Helena Garcia-Castro, Luis Baudouin-Gonzalez, Ruixun Wang, Naïra Sarkis, Alexander Günther Nikola, Venkata Sai Poojitha Koka, Nathan J. Kenny, Natascha Turetzek, Matthias Pechmann, Jordi Solana, Alistair P. McGregor

**Affiliations:** 1 Department of Biological and Medical Sciences, Oxford Brookes University, Oxford, OX3 0BP UK; 2 Department of Biosciences, Durham University, Durham, DH1 3LE UK; 3 Oxford University Museum of Natural History, University of Oxford, Oxford, OX1 3PW UK; 4 Institute for Zoology, Biocenter, University of Cologne, Zuelpicher Str. 47B, 50674 Cologne, Germany; 5 Evolutionary Ecology, Faculty of Biology, Biocenter, Ludwig-Maximilians-University Munich, Planegg-Martinsried, Germany; 6 Department of Biochemistry Te Tari Matū Koiora, University of Otago, Dunedin, New Zealand

**Keywords:** Spider, *Parasteatoda tepidariorum*, Single-cell RNA sequencing, Cell atlas, Development, Hox genes, Segmentation, Head patterning, Extra-embryonic

## Abstract

Spiders are a diverse order of chelicerates that diverged from other arthropods over 500 million years ago. Research on spider embryogenesis, particularly studies using the common house spider *Parasteatoda tepidariorum*, has made important contributions to understanding the evolution of animal development, including axis formation, segmentation, and patterning. However, we lack knowledge about the cells that build spider embryos, their gene expression profiles and fate. Single-cell transcriptomic analyses have been revolutionary in describing these complex landscapes of cellular genetics in a range of animals. Therefore, we carried out single-cell RNA sequencing of *P. tepidariorum* embryos at stages 7, 8 and 9, which encompass the establishment and patterning of the body plan, and initial differentiation of many tissues and organs. We identified 20 cell clusters, from 18.5 k cells, which were marked by many developmental toolkit genes, as well as a plethora of genes not previously investigated. We found differences in the cell cycle transcriptional signatures, suggestive of different proliferation dynamics, which related to distinctions between endodermal and some mesodermal clusters, compared with ectodermal clusters. We identified many Hox genes as markers of cell clusters, and Hox gene ohnologs were often present in different clusters. This provided additional evidence of sub- and/or neo-functionalisation of these important developmental genes after the whole genome duplication in an arachnopulmonate ancestor (spiders, scorpions, and related orders). We also examined the spatial expression of marker genes for each cluster to generate a comprehensive cell atlas of these embryonic stages. This revealed new insights into the cellular basis and genetic regulation of head patterning, hematopoiesis, limb development, gut development, and posterior segmentation. This atlas will serve as a platform for future analysis of spider cell specification and fate, and studying the evolution of these processes among animals at cellular resolution.

**Supplementary Information:**

The online version contains supplementary material available at 10.1186/s13227-024-00224-4.

## Introduction

Studying the embryology of arthropods, particularly insects, has helped to identify toolkit genes and their roles in development, and elucidated ancestral mechanisms of developmental regulation on one hand, and how these processes evolve on the other [1]. Chelicerates, including spiders, represent an outgroup to mandibulate arthropods. Studying their development provides a unique perspective to better understand the evolution of embryogenesis among arthropods and other animals [2].

**Fig. 1:**
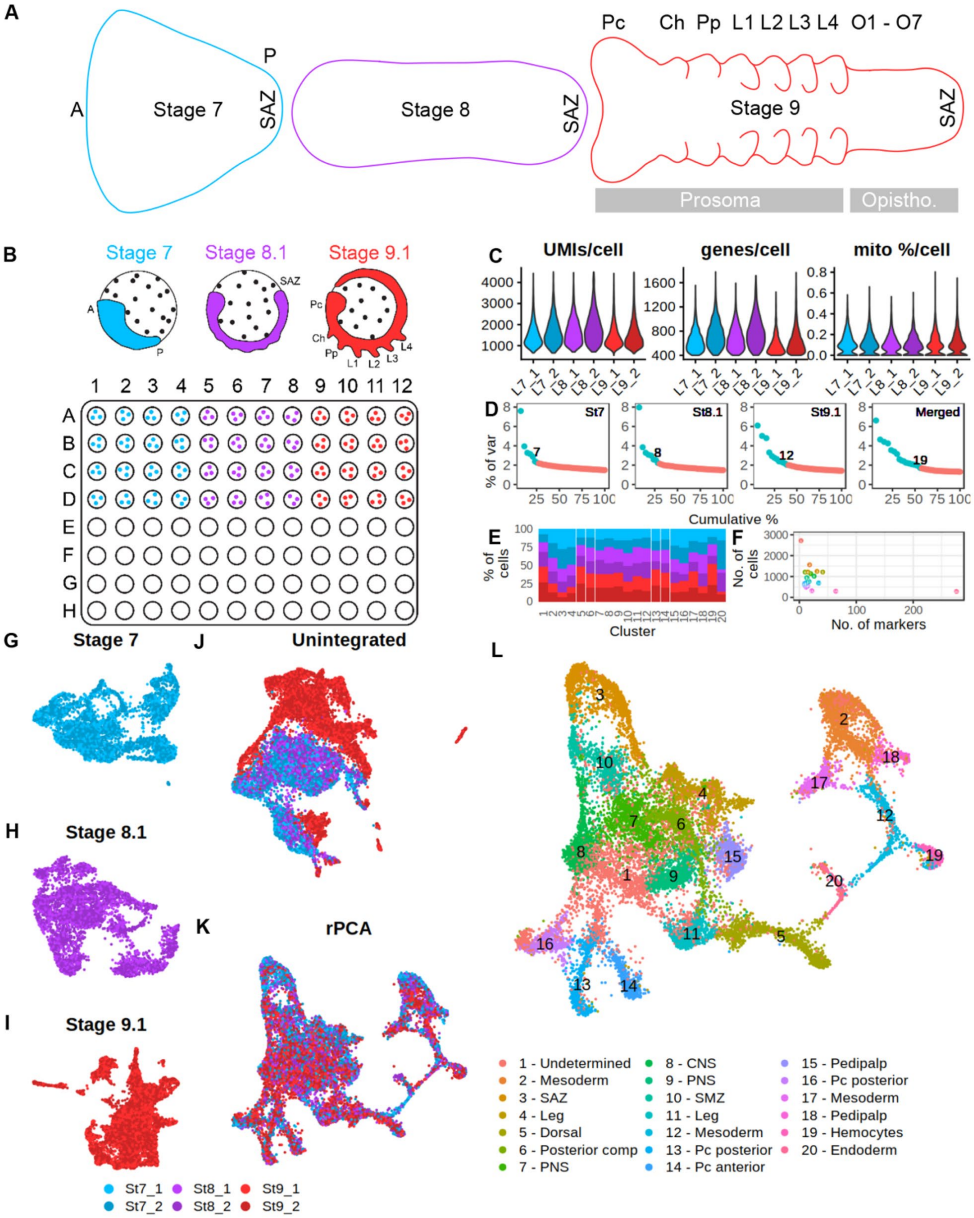
Single cell sequencing of three embryonic stages of *P. tepidariorum.* **A** Schematic of stages 7, 8.1 and 9.1 of *P. tepidariorum* embryos. Stage 7 has a fan-like shape with anterior and posterior poles, and has formed the segment addition zone (SAZ). Stage 8 extends the germband, and by stage 9 the prosomal limb buds are visible. **B** Stages 7, 8.1 and 9.1 were collected, dissociated with ACME, and cells from each dissociation were plated as shown for the first round of SPLiT-seq barcoding. **C** Metrics of UMI, genes and mitochondrial expression in each library. **D** Significant PCAs per stage and all stages merged show that the significant PCAs increase from stage 7 to 9.1, with the merged data containing the most. **E** Percentage of cells from each stage/library for each cluster, normalised by taking an equal number of random cells from each stage. **F** Association between number of cells and markers per cluster. **G**–**I** UMAP for each stage. **J** UMAP for all stages merged without integration and (**K**) with rPCA integration. **L** UMAP and cell clustering with annotation derived from ISH of marker genes. *A* anterior, *P* posterior, *Pc* precheliceral region, *Ch* cheliceral, *Pp* pedipalpal, *L1 to L4* leg-bearing 1 to 4, *O1 to O7* opisthosomal segments, *SAZ* segment addition zone, *SMZ* segment maturation zone, *PNS* peripheral nervous system, *CNS* central nervous system

The common house spider *Parasteatoda tepidariorum* has proven to be a powerful model for understanding the genetic regulation of key processes during spider embryogenesis and specific spider innovations [2–6]. *P. tepidariorum* embryos initially form a radially symmetrical germ disc in one hemisphere, with the extra-embryonic and yolk tissue in the other [6–10]. Radial symmetry is broken during embryonic stages 5 and 6 to form a germ band by stage 7 (Fig. 1A) [6, 11–13]. Therefore, generation of the bilaterally symmetrical germ band with both antero-posterior (A-P) and dorso-ventral (D-V) axes by stage 7 is a key point in embryogenesis (Fig. 1A). Subsequently, stages 7 to 9 encompass several important developmental events: *decapentaplegic* (*dpp*)/*short gastrulation* (*sog*) mediated patterning along the D-V axis, and Hox instructed segment identity along the A-P axis (Fig. 1A) [8, 14, 15], the germ layers begin to differentiate into the corresponding[16] tissues and organs [16, 17], the head forms and neurogenesis begins [8, 18–20], the prosomal (cephalothorax) segments form [12, 21–23], concomitant with formation and growth of the limb buds [2, 6, 24] and the opisthosomal (abdominal) segments are added sequentially posteriorly from the segment addition zone (SAZ) (Fig. 1A) [17, 25, 26]. Therefore, embryonic stages 7 to 9 see both linear changes in cell states as they differentiate into growing structures and tissues, and reiterative regulatory processes to generate the body along the A-P axis.

To better understand these processes, we require differential gene expression data at cellular resolution during these stages of embryogenesis. Single-cell RNA sequencing (scRNA-seq) transcriptomics can provide this to allow cell state characterisation and differentiation, and unbiased identification of key marker genes [24, 27–30]. Indeed, scRNA-seq has successfully been applied to embryos of a rapidly growing number of animals [31–38], including stage 5 and 6 *P. tepidariorum* embryos, which identified cells corresponding to the three germ layers and reconstructed A-P polarity and initial patterning based on known and new markers genes [39], as well as recent single cell analysis of embryonic stages 10 to 12 for this spider [40].

To better understand spider embryogenesis, we carried out scRNA-seq at stages 7, 8 and 9 taking advantage of our recent advances in cell dissociation, combining ACetic-MEthanol (ACME) dissociation [29] and SPLiT-seq scRNA-seq [41] technology. While most enzymatic dissociation methods process live cells, which can incur damage and stress-related transcriptional signatures, ACME circumvents these problems by fixing cells during the dissociation process. The stages profiled are separated by just a few hours of developmental time, and ACME crucially fixes those stages avoiding hours of live cell dissociation where the cells may keep their developmental process ex vivo. Additionally, many droplet-based methods are sensitive to the introduction of noise from ambient RNA and cellular debris, which can also falsely increase their gene and UMI per cell counts. In contrast SPLiT-seq minimises these issues because ambient RNA is eliminated with the supernatant in each of the successive centrifugation steps for each of the split-pool rounds. Furthermore, the introduction of a FACS step immediately prior to cell lysis eliminates cellular debris. Therefore, while SPLiT-seq usually results in lower gene and UMI per cell counts, compared to some other technologies, this approach resolves clusters that are robust to clustering conditions and subsampling.

Our analysis of stage 7, 8 and 9 spider embryos combining ACME dissociation and SPLiT-seq scRNA-seq allowed us to define cell types and capture new genes involved in several important developmental processes during these key embryonic stages, including germ layer differentiation, axial patterning, head and CNS development, and limb development as well as new perspectives on the role of the so called ‘extra-embryonic’ cells. Furthermore, our results provided new insights into the regulation of the reiterative formation of the opisthosomal segments, most of which are sequentially generated from a posterior SAZ during stages 7, 8 and 9.

## Results

### Single-cell sequencing of three stages of spider embryogenesis

To better understand cell states during spider embryogenesis we sequenced single-cells from three embryonic stages (7, 8.1 and 9.1) of *P. tepidariorum* (Fig. 1B) [6]. We focused on these stages because they mark the onset and/or continued progress of key developmental processes, including segmentation, and we lack information about the cells and their expression profiles during these processes [5, 6, 8, 5–6, 14, 5–6, 5–6, 5–6].

We carried out separate ACME dissociations for each stage [29]. These dissociation samples were subjected to SPLiT-seq barcoding [41], processing all stages in parallel (Fig. 1B). Cells from each stage were barcoded separately so that the first barcode could be used for de-multiplexing stages (Fig. 1B). After the third round of SPLiT-seq barcoding, FACS was used to sort cells into two samples for the fourth round of barcoding to generate two sequencing libraries. These libraries, therefore, constitute different cells from the same dissociation that had different sequence indexes, and were pooled and sequenced on the same Illumina lane. A total of 481,741,227 raw sequence reads were generated from these two libraries (Additional file 3: Table S1). After trimming and barcode assignment using DropSeq tools, 328,915,611 (68%) reads remained (Additional file 3: Table S1). Star mapped 82% of input reads to a new *P. tepidariorum* annotation that was constructed to maximise 3ʹ completeness including UTRs to improve mapping (Additional file 3: Table S1). From these mapped genic reads, a total of approximately 30,000 cell transcriptomes were captured with a minimum of 100 genes, prior to Seurat filtering and doublet removal. Nearly all cells had < 1% mitochondrial gene expression, indicating minimal transcriptional noise from cell stress in the dataset (Fig. 1C) consistent with the use of ACME. After filtering (see Materials and Methods) based on UMI counts, genes expressed per cell, mitochondrial expression and doublet removal, the total processed dataset contained 18,516 cells, with 14,370 (43%) genes expressed out of a total 33,413 genes that we re-annotated, to attain UTR annotations for mapping, in *P. tepidariorum*. Stages 7, 8.1, and 9.1 were represented by 4824, 4833, 8859 cells, respectively, with median UMI count per cell of 1465, 1656, and 1343, and a median of 674, 713, and 563 genes quantified per cell (Fig. 1C and Additional file 3: Table S1). Stages 7 and 8 were comparable in these metrics, whereas stage 9.1 had fewer UMI and gene counts per cell, but more cells overall (Fig. 1C and Additional file 3: Table S1). For all datasets, UMIs and genes per cell per cluster were reasonably similar except for the first cluster, which often exhibited lower UMI that other clusters in each dataset (Additional file 3: Fig. S1).

Merging of cells from the two libraries for each stage and Seurat processing showed that libraries of each stage were comparable (Fig. 1G–I). Cells from each library were distributed across UMAPs for each stage, with clusters containing cells from both libraries, suggesting no issues during the fourth round of barcoding and library preparation. Processing of each stage separately revealed an increase in the contribution of informative principal components increasing from 7 to 8 and 12 for embryonic stages 7, 8.1 and 9.1, respectively (Fig. 1D). This suggests, as expected, that transcriptomic complexity increased as development progressed. Markers from each stage were identified using an in-cluster-versus-all-others, identifying 130, 117, and 230 markers for stages 7, 8.1 and 9.1, respectively. All markers are provided in Additional file 1. Additionally, we have made available the fully processed datasets of each stage separately and merged datasets, along with a markdown and HTML of plots for all genes discussed in this study (https://doi.org/10.6084/m9.figshare.24899643.v1).

### Stage sample integration

We assessed all stages together by merging and processing them without integration. This showed that stage 9.1 differed from stages 7 and 8.1 because there were clusters containing only/mostly stage 9.1 cells (Fig. 1J). This suggested that: (1) there may be large differences between samples due to independent dissociations of embryos from each stage; (2) or that the greater number of cells from stage 9.1, and lower median UMI and gene counts, cause biologically similar cell states to appear transcriptionally different; (3) or that there are real biological transcriptional signatures at stage 9.1 causing cells not to be clustered with stage 7 and 8.1. Note the time interval between stage 7 and 8.1 (up to 14 h) is much shorter than between stage 8.1 and 9.1 (up to 24 h), which might explain the separation of stages [6].

We, therefore, assessed different integration approaches and explored their impact on cluster markers from the three stages by comparing the results to the unintegrated data. Since integration can force cell states to appear more comparable, information from stage 9.1 might be lost during the integration, and cause exclusion of stage 9.1 specific marker genes.

For integration we used both CCA and rPCA from Seurat, as well as Harmony [52]. We performed the same pre-processing, variable gene selection and normalisation prior to integration. Seurat rPCA, CCA and Harmony produced similar results, with only Harmony appearing to not integrate stage 9.1 as strongly as rPCA/CCA (Additional file 3: Fig. S2).

To establish whether integration generated data artefacts, or unlikely clustering patterns, we iterated through integration anchors (5–45) with the rPCA method, which affects the strength of integration, using a range of residual variance cut-off (1.2–1.7) (Additional file 3: Fig. S3, S4). Quantification of clustering similarity using an adjusted RandIndex [53] showed that the threshold of variable genes had the most effect on clustering similarity, and that integration with 30 to 40 anchors were most stable (Additional file 3: Fig. S4). Therefore, we proceeded with rPCA integration with 1.3 threshold for variable genes and 40 anchors to assess all stages together.

To determine clusters in the integrated data we estimated a stable clustering resolution given the lack of information regarding spider cell type diversity. This approach revealed 20 clusters that were represented by cells from all stages/samples (see Materials and Methods) (Fig. 1K, L), but still showed variability in the abundance of cells from a given stage within each cluster (Fig. 1E), and cluster sizes ranged from 2719 (14.7%) cells in cluster 1, to 283 (1.5%) cells in cluster 20 (Fig. 1F).

Marker genes for the 20 clusters were predicted with an in-cluster-versus-all-others approach, including genes that were expressed in at least 25% of cells in their respective cluster and an adjusted *p*-value return threshold of 1e-5. A total of 491 genes were identified as cluster markers, with numbers of markers per cluster ranging from 3 (cluster 1) to 276 (cluster 20) (Fig. 1F). Cluster markers for integrated and stage data are provided in Additional file 1.

Given that the clustering showed sufficient structure and information we then interrogated cell clusters and characterized marker genes during these three stages with respect to key developmental processes. To assess whether stage-specific clusters were well-represented in the merged datasets we compared stage-specific cluster markers to unintegrated and integrated (rPCA) marker lists with a hypergeometric distribution test. We also used ClusterMap [54] to similarly compare cluster markers between datasets. Overall, we detected clear signatures that the all merged datasets possessed clusters that were also mostly represented in stage-specific clusters (Additional file 3: Fig. S5–S7). However, while there were some differences i.e., Harmony and unintegrated datasets were more comparable to each other than between CCA and rPCA, as suspected from unintegrated versus integrated data, we identified that stages 7 and 8.1 had most of their markers present in the integrated marker lists, whereas stage 9.1 had several dozen markers missing (Additional file 3: Fig. S7). The majority of stage 9.1 cluster markers that were not present in merged datasets correspond to cells in cluster 19 of the rPCA integrated marker list both in terms of marker list overlap and the spatial expression of markers (Additional file 3: Fig. S8). To circumvent the issue that each of these datasets might have specific markers not present in others, we selected markers that were predominantly found in all merged datasets to capture the most robust signals of cellular transcriptional identity. Therefore, integration helped to merge stages without considerable loss of stage-specific information.

### Clusters with the greatest G1 phase ratio relate to cells from gut, dorsal, and mesoderm lineages

Many new cells are required to build the differentiating germ layers, tissues, and organs during stages 7 to 9 of *P. tepidariorum* embryogenesis. We, therefore, asked whether any of the cell clusters were associated with signals of enhanced cell division and what tissues they may contribute to. We identified orthologs of *Drosophila melanogaster* genes that are associated with G1, S, G2/M phases of the cell cycle and quantified their expression in clusters using Seurat cell cycle scoring.

**Fig. 2:**
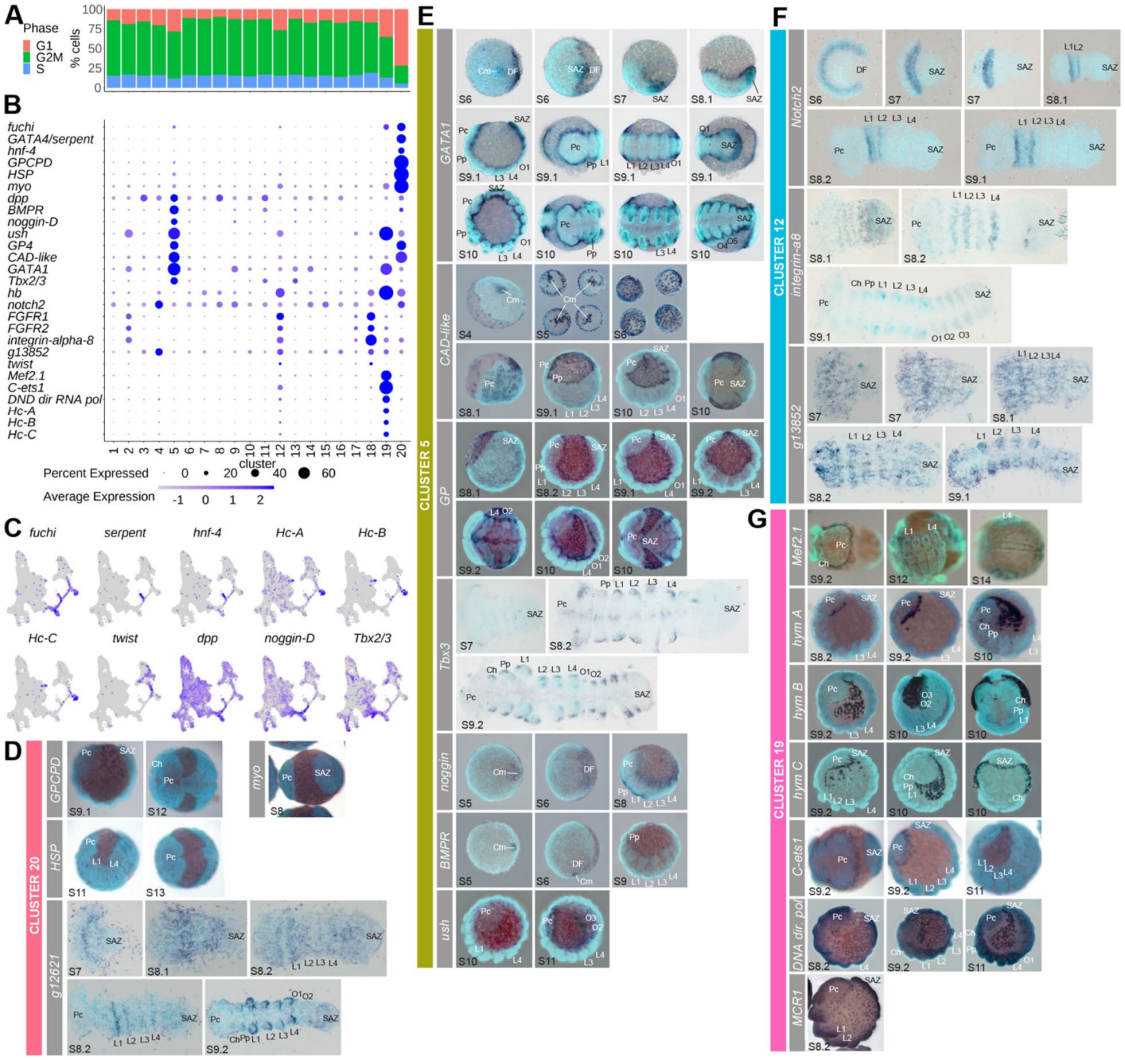
Cell cycle differences reveal four clusters with distinct endodermal and mesodermal characteristics. **A** Cell cycle gene scoring shows four clusters, 5, 12, 19 and 20, with more than 25% G1 phase cells (red). **B** Dotplot of marker genes. **C** UMAPs of some markers and non-markers previously identified to have similar expression as markers expressed in clusters 5, 12, 19 and 20. **D**–**G** Spatial expression of marker genes during embryogenesis assayed by in situ hybridisation. *Pc* precheliceral region, *Ch* cheliceral, *Pp* pedipalpal, *L1 to L4* leg-bearing 1 to 4, *O1 to O12* opisthosomal segments, *SAZ* segment addition zone, *DF* dorsal field, *Cm* cumulus

Sixteen clusters had similar proportions of each cell cycle phase, however the G1 phase was found in over 25% of cells in clusters 5, 12 and 19, and approximately 75% for cluster 20 (Fig. 2A). This suggests that these four clusters exhibit different proliferation dynamics from other clusters, although none of the cell cycle genes were markers of these four clusters, or any other clusters. We next assessed the embryonic expression of markers from these four clusters.

Cluster 20 contained the highest number of significant marker genes (Fig. 1F). We found that cluster 20 was marked by the recently characterized GATA gene *fuchi* [*g16870*], which is expressed in the endoderm [55]. These cells also expressed *hepatocyte-nuclear factor-4* (*hnf-4*) [*g4057*] and *serpent*/*GATA4* [*g7067*], which are also expressed in the endoderm, and although they were not significant markers of cluster 20 in the rPCA integrated dataset, they were both markers of cluster 16 in the Harmony integrated dataset. (Fig. 2B, C) [49, 55]. We analysed three further markers of cluster 20 *GPCPD* [*g1958*], *HSP* [*g14898*] and *g12621* (Fig. 2B–D). Like *fuchi*, *serpent* and *hnf-4*, all three marker genes were expressed in extra-embryonic cells that contribute to the endoderm (Fig. 2D). However, *g12621* was also expressed in the mesodermal cells in the germband. Additionally, *GPCPD* was previously used as an endoderm marker, and this gene is expressed earlier in the peripheral cells of the germdisc and in the cumulus [12] (a group of cells that migrates during stage 5 to break radial symmetry and determine the dorsal field at stage 6 [8, 9, 12]), further evidencing that this cluster relates to endoderm cells.

Cluster 5 was marked by the dorsal determinant *dpp* [*g29377*] [8], as well as *BMPR* [*aug3.g2323*], *noggin-D* [*g27229*], *ush* [*aug3.g16893*], *platelet glycoprotein 4* (*GP4*) [*g4985*], *CAD-like* [*g6385*], *GATA1* [*g4744*] [55] and *Tbx2/3* [*aug3.g3745*] (Fig. 2B, C and E). Like *dpp*, genes such as *BMPR*, *CAD-like, noggin-D* and *GATA1* were all expressed in the cumulus, indicating that cluster 5 cells might originate from the mesenchymal cumulus cells underneath the epithelial cells and have dorsal identity [8, 9, 23, 39]. Indeed, several of these markers (*noggin-D*, *GATA1* and *BMPR*) were present in the cumulus cell cluster in the recent single-cell analysis of stage 5 embryos [39]. We found that *GATA1*, *BMPR, ush*, *GP4* and *noggin-D* were all expressed in the dorsal field at stage 6 (Fig. 2E). *noggin-D* and *GATA1* were also expressed from stages 7 to 9 broadly around the dorsal periphery of the germband. Previous analysis showed that the embryonic expression of *GATA1* borders ventral *sog* expression at stage 8 [12]. However, *BMPR* expression was restricted to dorsal domains in each appendage at stages 7 to 9 (Fig. 2E). *Tbx2/3* was expressed in the precheliceral region and at the ventral midline, as well as in dorsal regions of prosomal appendages, like *BMPR* (Fig. 2E). We observed that in addition to the expression of *CAD-like* in the cumulus, this gene was also expressed in large cells surrounding the germdisc and in the extra-embryonic region at stage 5 (Fig. 2E). Subsequently, *CAD-like* expressing cells at stage 6 were present in the dorsal field and extra-embryonic region, and at stages 7 to 8 beneath the germband. At stage 9, expression of this gene is present in extra-embryonic cells around the germband, not rather than underneath the germband, like all other cluster 5 markers surveyed (Fig. 2E). These results are consistent with cluster 5 cells originating from the cumulus and  representing cells in the extra-embryonic region.

Cluster 12 was marked by *hunchback* (*hb*) [*g27583*], which was previously shown to be necessary for development of the L1, L2 and L4 prosomal leg-bearing segments (Fig. 2B) [44]. Another cluster 12 marker, *Notch2* [*g30344*], was first expressed in a single broad domain in the anterior of the germdisc at stage 6 that splits during stage 7 to 8 and, like *hb*, was subsequently expressed in L1 and L2 from stage 8 (Fig. 2F) [44]. Cluster 12 was also marked by the mesodermal genes, *FGFR1* [*g11749*] and *FGFR2* [*g3961*] [13], as well as *integrin-alpha-8* [*g23098*] and an uncharacterized gene *g13852,* which were also expressed throughout the mesoderm of the prosoma and opisthosoma (Fig. 2F). In a previous study, *integrin-alpha-8* was also identified as a marker for a cluster at stage 5 and was expressed in cells that form a mesodermal cell lineage at the germdisc periphery [39]. While *twist* [*g22789*], a mesodermally expressed gene in *P. tepidariorum*, was not a marker of cluster 12, it was expressed in cells of clusters 2, 12, 17, and 18 (Fig. 2C). This suggests that cluster 12 represents mesoderm that originates from cells at the germ disc periphery but later become broadly distributed across the germband.

Cluster 19 was marked by the mesodermal gene *Mef2.1* [*g3542*] (Fig. 2B and G) [56, 57]. *Mef2.1* and three other markers, *hemocyanin A* (*Hc-A*) [*g11873*], *C-ets1* [*g472*], and *DNA directed RNA pol* [*g17128*], all showed expression from the dorsal regions around the head into the extra-embryonic region (Fig. 2G). Two other hemocyanin genes (*hemocyanin B* [*g13621*] and *hemocyanin C* [*g22680*]) that were markers of stage 9.1, cluster 11, which had the best marker overlap with cluster 5 from the integrated data (Additional file 3: Fig. S7), had similar expression to *hemocyanin A* (Fig. 2G). The post stage 9 expression of these marker genes suggests either dorsal cells are migrating across the extra-embryonic region earlier than dorsal closure at stage 13 [6], or that extra-embryonic cells are being recruited to dorsal tissues of the embryo proper. Collectively, given the function of the orthologous genes in *D. melanogaster* [58–61], cluster 19 cells potentially correspond to hemocytes, which until now have not been identified in spiders.

Overall, we were able to characterize endodermal and mesodermal cell populations, including a newly identified potential hemocyte-related population. Our dataset will serve as a resource for further characterising these cell populations during embryogenesis.

### Hox markers are consistent with A-P cell identity and evidence sub- and/or neo-functionalisation

*P. tepidariorum* has retained two Hox gene clusters following the whole genome duplication (WGD) event in an ancestor of arachnopulmonate arachnids and is missing only a second copy of *fushi tarazu* (*ftz*) [14]. During stages 7 to 9, the Hox genes are generally expressed in a collinear fashion across the A-P axis of *P. tepidariorum* embryos [14] and, therefore, we assessed the expression of these key patterning genes in the scRNA-seq data.

**Fig. 3:**
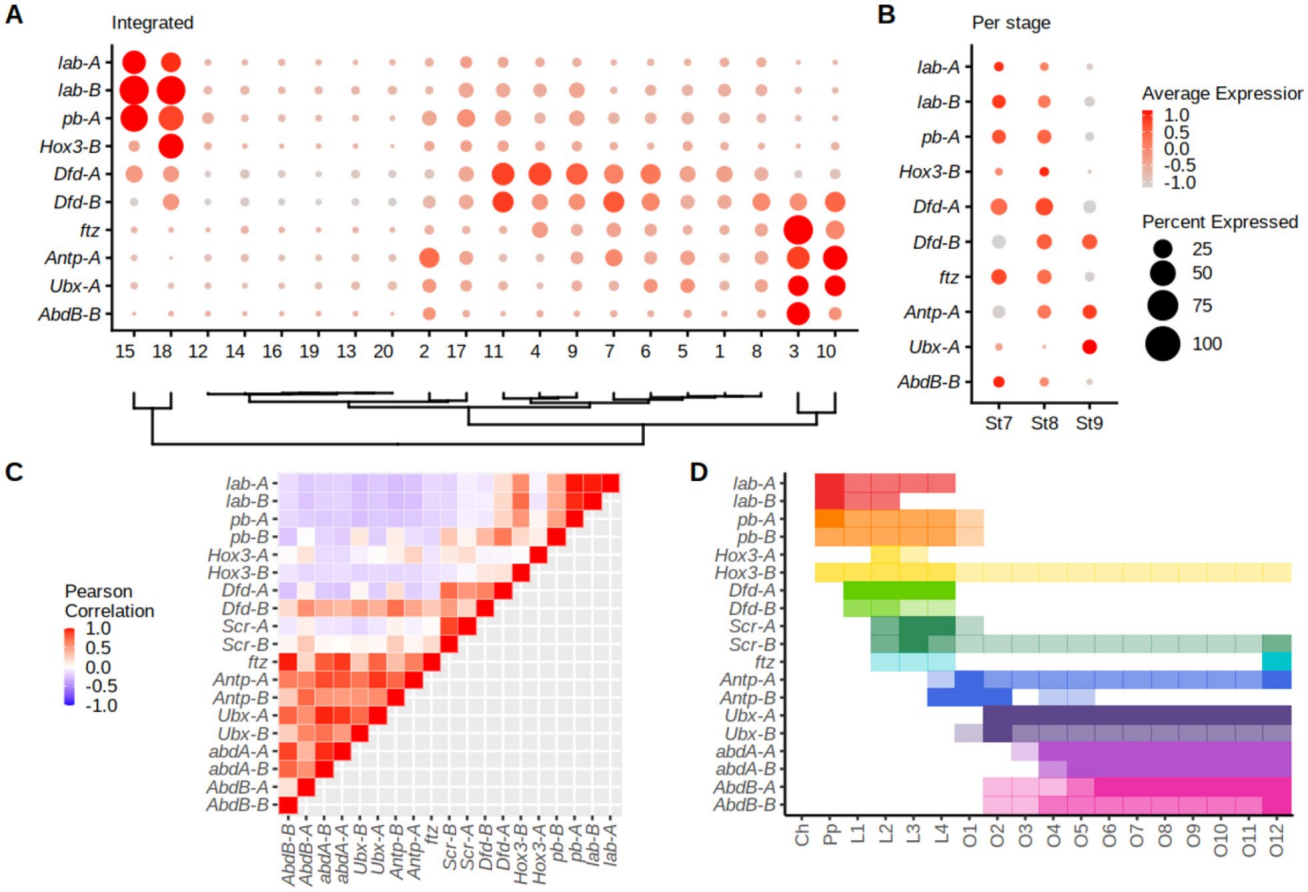
Hox expression in scRNA-seq data. **A** Dotplot of the expression of all ten Hox markers in cell clusters ordered hierarchically for integrated data. **B** Temporal expression of thirteen Hox markers across the three stages. The legend in (**B**) relates to the expression and number of expressing cells per cluster in both (**A)** and (**B**). **C** Pearson’s correlation coefficients of SCT normalized average Hox expression across all 20 clusters reveal three cluster types related to pedipalpal, leg-bearing and opisthosomal identities. **D** Positioning of Hox expression across the A-P axis of *P. tepidariorum*. Spatial expression intensity (detected by RNA in situ hybridisation) reflected using opaque (strong expression) to more transparent colouring (weak expression). Adapted from Schwager et al. 2017, with the addition of new *Hox3-A* expression data (Additional file 3: Fig. S9D). *Ch* cheliceral, *Pp* pedipalpal, *L1-L4* leg-bearing 1 to 4, *O1-O12* opisthosomal segments 1 to 12

Expression of all nineteen Hox genes in *P. tepidariorum* was detected in the scRNA-seq data and ten were markers of eight clusters (clusters 2, 3, 4, 9, 10, 11, 15, 18) of the integrated data (Fig. 3A and Additional file 3: Fig. S9A, B). Fitting with the collinear expression principle, the posterior Hox genes were expressed at stage 9.1 more so than stages 7 and 8.1 (Fig. 3B). Hierarchical clustering of our single cell data using Hox marker expression identified six groups (Fig. 3C, D). Four of these corresponded to spatial regions of the spider embryo. Whereas the others potentially represent one group of spatially distributed cells expressing but not marked by Hox genes and another group of non-Hox expressing cells (Fig. 3C, D) [14, 15].

The four groups that relate to spatially confined Hox expression suggest that some clusters represent cells that are restricted to segments across the A-P axis. One group related to the pedipalpal segment and contained clusters 15 and 18, which are marked by *labial-A, labial-B,* and *proboscipedia-A*, which are most highly expressed in the pedipalpal region (Fig. 3C, D) [14, 15]. Another group relating to prosomal segments contained clusters 4, 9 and 11, marked by *Deformed-A* and *Deformed-B*, which are exclusively expressed in the leg-bearing segments (Fig. 3C, D) [14]. Additionally, other markers of cluster 11 included *Distal-less* (*Dll*) [*g10793*] [45] and *sp6-9* [*g22966*], and a previously unanalyzed marker *basonuclin* [*g29744*], which are also expressed in leg-bearing segments (Additional file 3: Fig. S9C) [62, 63], further supporting that these clusters contain cells that are found in prosomal segments. The other two groups relate to the opisthosomal region, with one group containing clusters 3 and 10, marked by *ftz*, *Antennapedia-A*, *Ultrabithorax-A* and *Abdominal-B-B*, which are all expressed in the opisthosoma, and another group containing cluster 2, marked only by *Antennapedia-A* (Fig. 3C, D) [14].

The remaining two groups comprised of clusters that did not have Hox markers. One of them, which contained clusters 1, 5, 6, 7 and 8, showed expression of many Hox genes but none passed statistical thresholds to become markers, suggesting that these cell clusters represent cells that are distributed across the A-P axis (Fig. 3C, D). The other group contained clusters 12, 13, 14, 16,19, and 20, which in contrast did not express any Hox gene, suggesting that there are populations of cells in *P. tepidariorum* embryos that are not patterned by Hox genes at stages 7 to 9.1 (Fig. 3C, D).

We next assessed the correlation of Hox expression across clusters to see if ohnologs show similar or divergent expression across clusters (Fig. 3C). Generally, this supported the hierarchical clustering showing distinct correlation groups relating to pedipalpal, leg-bearing and opisthosoma identities (Fig. 3C, D). It also provided further evidence for the hypothesis that some Hox ohnologs have undergone sub- and potentially neo-functionalisation previously indicated by comparing spatial and temporal expression pattern analysis. *Hox3* ohnologs, for example, have low correlation (r = − 0.007), whereas other Hox ohnologs have highly correlated expression across clusters e.g., *labial* (r = 0.97). Another example is the two *proboscipedia* (*pb*) ohnologs, which are both expressed in mesoderm of appendages, but only *pb-A* is expressed strongly in the pedipalpal segment [14]. This pattern is also reflected in the scRNA-seq data, whereby *pb-A* is a marker (of clusters 15 and 18), whereas *pb-B* is not a marker of any cluster and only expressed lowly in several prosomal regions. Furthermore, *Sex combs reduced-A* (*Scr-A*) is expressed mostly in leg-bearing segments, whereas *Scr-B* is also expressed in the SAZ [14] consistent with expression detected in cells with opisthosomal identity in the scRNA-seq data (Additional file 3: Fig. S9B).

Overall, our analysis of Hox gene expression showed that the scRNA-seq data captured key developmental transcription factors and allowed us to compartmentalise many cell clusters into broad regions of the body plan, as well as clusters that were mostly void of Hox expression. Furthermore, our data support previous expression pattern analysis that spider Hox ohnologs have undergone sub- and potentially neo-functionalisation after WGD [14] and offers a resource for future investigations of other ohnologs.

### Patterning of the spider precheliceral region

**Fig. 4:**
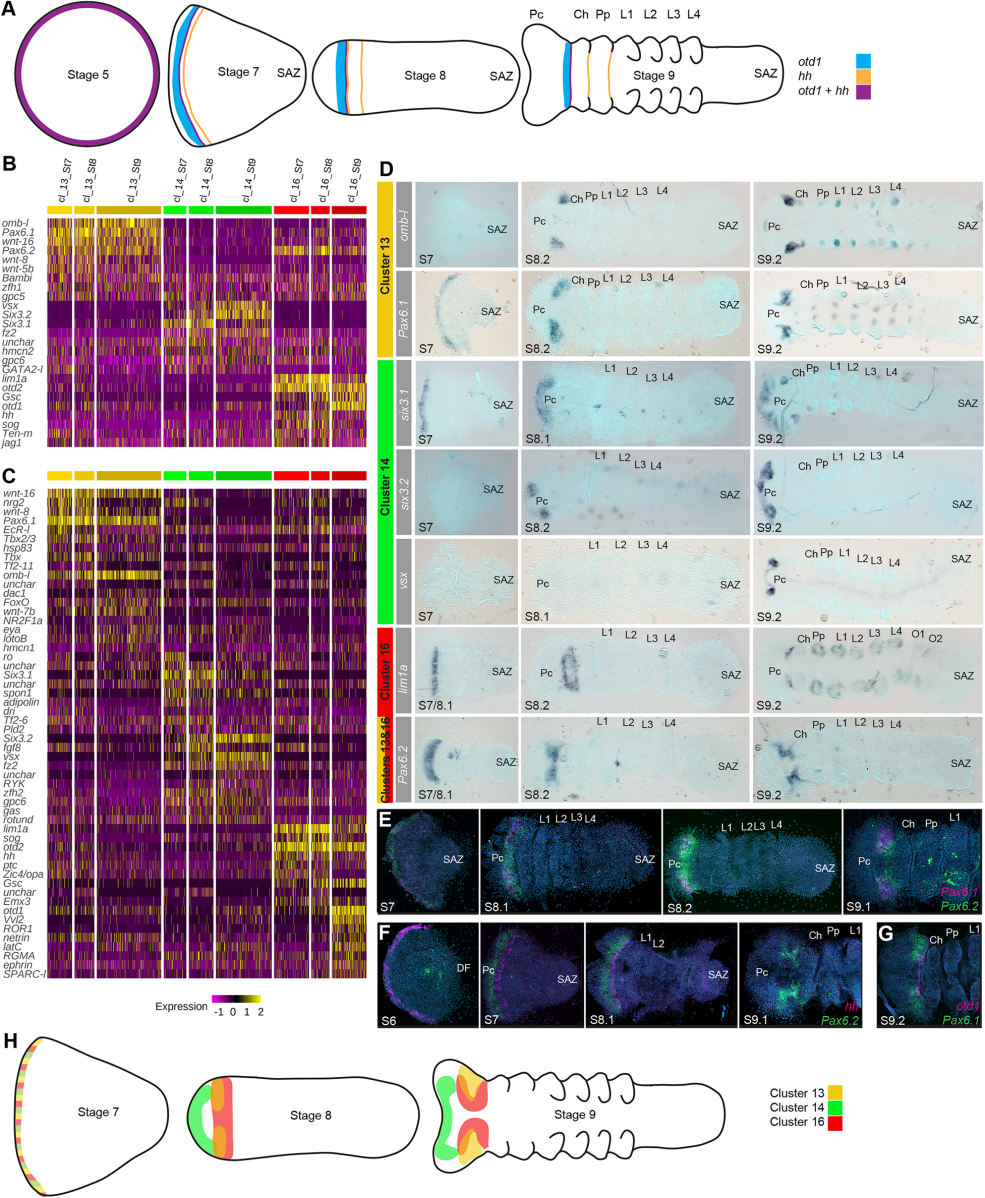
Clusters contributing to the spider precheliceral region. **A** Schematic overview of *otd-1* and *hh* expression during prosomal patterning. At stage 5 *otd-1* and *hh* expression overlaps. At stage 7 expression of both genes migrates posteriorly from the anterior rim, and *hh* expression splits to form the presumptive pedipalp segment. At stage 8, a second splitting of *hh* expression forms the presumptive cheliceral segment. At stage 9 *otd-1* and *hh* are expressed at the posterior region of the pre-cheliceral region [22, 46]. **B** Heatmap of the top ten markers for clusters 13, 14 and 16 relating to pre-cheliceral region patterning. **C** Heatmap of the top ten markers of the same clusters but also comparing between stages. These show some staggering of markers across stages, suggesting possible differentiation pathways **D** Expression of marker genes, colour bars represent the clusters they are associated with relative to Figures **B** and **C**. **E**–**G** Double fluorescent in situ hybridisation of markers. **E** *Pax6.1* and *Pax6.2* markers relate to cells from clusters 13 and 16, which show expression moving from the anterior rim towards the posterior of the pre-cheliceral region. *Pax6.1* expression is more dorsal and anterior compared to *Pax6.2*, with regions of non-overlapping expression (**F**). The most posterior expression of *Pax6.2* co-expresses *hh* (**H**) Overview of the location of proposed three head patterning clusters in the scRNA-seq data from stage 7 to 9. *Pc* pre-cheliceral region, *Ch* cheliceral, *Pp* pedipalpal, *L1 to L4* leg-bearing 1 to 4 segments, *SAZ* segment addition zone

The *P. tepidariorum* germdisc periphery represents the future anterior of the germband that later gives rise to the pre-cheliceral region as well as structures like the brain, mouth parts and eyes [18, 19, 46]. *hedgehog* (*hh*) and *orthodenticle-1* (*otd-1*) are co-expressed at the germdisc periphery at stage 5 and begin to move posteriorly at stage 6 (Fig. 4A) [15, 22, 46]. The most posterior domain of *hh*, which does not co-express *otd-1*, splits during stages 7 and 8 to first generate the *lab-A* expressing pedipalpal segment, and then the cheliceral segment (Fig. 4A) [15, 22, 46]. However, the region of *hh* that co-expresses *otd-1* denotes the boundary of this *hh* domain and, therefore, represents the posterior boundary of the pre-cheliceral region (Fig. 4A). While *hh* and *otd-1* are involved in setting up the pre-cheliceral region it is unclear how the pre-cheliceral structures are thereafter patterned [46]. By assessing new and existing gene expression studies of cluster markers and per-stage-per-cluster markers, we identified three cell clusters, 13, 14 and 16, with different gene expression regionalisation and dynamics in the pre-cheliceral region. These three clusters were marked by known anteriorly expressed genes, *otd-1* and *hh*, but lacked Hox markers (Figs. 2 and 4B, C), consistent with previous observations that the pre-cheliceral region does not express Hox genes after the pedipalpal and cheliceral segments have been defined.

*otd-1* [*g5047*] and *hh* [*g23071*] were markers of cluster 16 (Fig. 4B, C), suggesting this cluster relates to cells of the pre-cheliceral region. We, therefore, analyzed two other markers from cluster 16 (Fig. 4B) and found that *lim1a* [*g12191*] and *Pax6.2* [*g12868*] were both expressed at stage 7 several cells posterior to the very anterior rim of the germband (Fig. 4D). Like *otd-1* the expression of *lim1a* and *Pax6.2* subsequently migrates more posteriorly, resulting in a broad band at the posterior boundary of the pre-cheliceral region at stage 8 (Fig. 4D). By stage 9 this band becomes restricted to two domains lateral to the ventral midline (Fig. 4D). This is consistent with another cluster 16 marker *sog* [*g13327*], which is expressed at the ventral midline [8] and in stage 7 and 8 cluster 16 cells but has reduced expression by stage 9 (Fig. 4C).

We assessed the cluster 13 markers *Pax6.1* [*g12873*], an *optomotor-blind* like gene [*aug3.g3790*], and *Tbx2/3*, which was also a cluster 5 marker (note that *Pax6.2* was also a cluster 13 marker – see above). *Pax6.1* and *Tbx2/3* were expressed earlier than the other cluster 13 markers. At stage 7, *Pax6.1* was expressed in a stripe along the anterior rim, and *Tbx2/3* had faint expression at the lateral edges of the germband close to the anterior rim (Fig. 4D). Their expression subsequently migrated towards the posterior of the pre-cheliceral region (Fig. 4D). However, in contrast to cluster 16 genes, most markers became limited to the posterior dorsal region of the pre-cheliceral region by stage 9 (Fig. 4D).

Cluster 14 cells were not marked by *otd-1* expression, however, we analyzed three markers, *six3.1* [*g1245*], *six3.2* [*g25543*], and *visual system homeobox (vsx)* [*aug3.g27186*], which were all expressed in the pre-cheliceral region (Fig. 4D). *six3.1* was initially expressed along the anterior rim of the germband at stage 7, while *six3.2* appeared at the anterior rim at stage 8, and finally *vsx* by stage 9 (Fig. 4D). All three genes (*six3.1*, *six3.2* and *vsx*) maintained their expression at the anterior of the pre-cheliceral region (Fig. 4D).

Double fluorescent in situ hybridisation of pre-cheliceral region markers was performed to better distinguish the expression dynamics and mutually exclusive regions between clusters. *Pax6.1* (cluster 13) was initially maintained at the anterior limit of the *Pax6.2* (cluster 13 and 16) expression domain (Fig. 4E). By stage 9.1 *Pax6.2* was more ventrally restricted and *Pax6.1* was more dorsally restricted, although their expression overlapped by a few cells, corroborating their distinct D-V domains of expression in the developing pre-cheliceral region detected by single in situ hybridisation (Fig. 4E). This D-V arrangement was also supported by the expression of *sog*, which is expressed ventrally [8], in cluster 16, but not in clusters 13 or 14 (Fig. 4B, C). *Pax6.2* remained anterior to the *hh* stripe from stage 7 to 8.1 and at stage 9.1 their expression overlapped at the posterior border of the pre-cheliceral region (Fig. 4F). This was further supported posterior *Pax6.1* expression overlapping with *otd-1* (Fig. 4G).

Overall, our data revealed new details of transcription factor expression in the developing pre-cheliceral region. This suggests that distinct combinatorial expression domains mark different regions of the pre-cheliceral region such as the forebrain, and hindbrain primordia (clusters 13 and 16), which become more obviously regionalised during stages 8 and 9 (Fig. 4H) [64, 65]. However, it is difficult to spatially place these cells across time from stage 7, since it is unknown whether gene expression is dynamic across cell fields, or if cell movement/recruitment underlie spatial changes in gene expression. Identification of markers between stages of each cluster in a per-stage-per-cluster approach reveals that all three clusters have transcriptional transitions between stages, suggesting there is dynamic expression (Fig. 4H). Future work will reveal how these expression dynamics relate to differentiation gene regulatory networks in pre-cheliceral patterning. Additionally, clusters 13, 14 and 16 had better overlap with clusters from stage 8.1 and 9.1 specific clustering, rather than with stage 7, consistent with the pre-cheliceral region being established after stage 7.

### Ventral midline patterning and diversity in peripheral nervous system cells

**Fig. 5:**
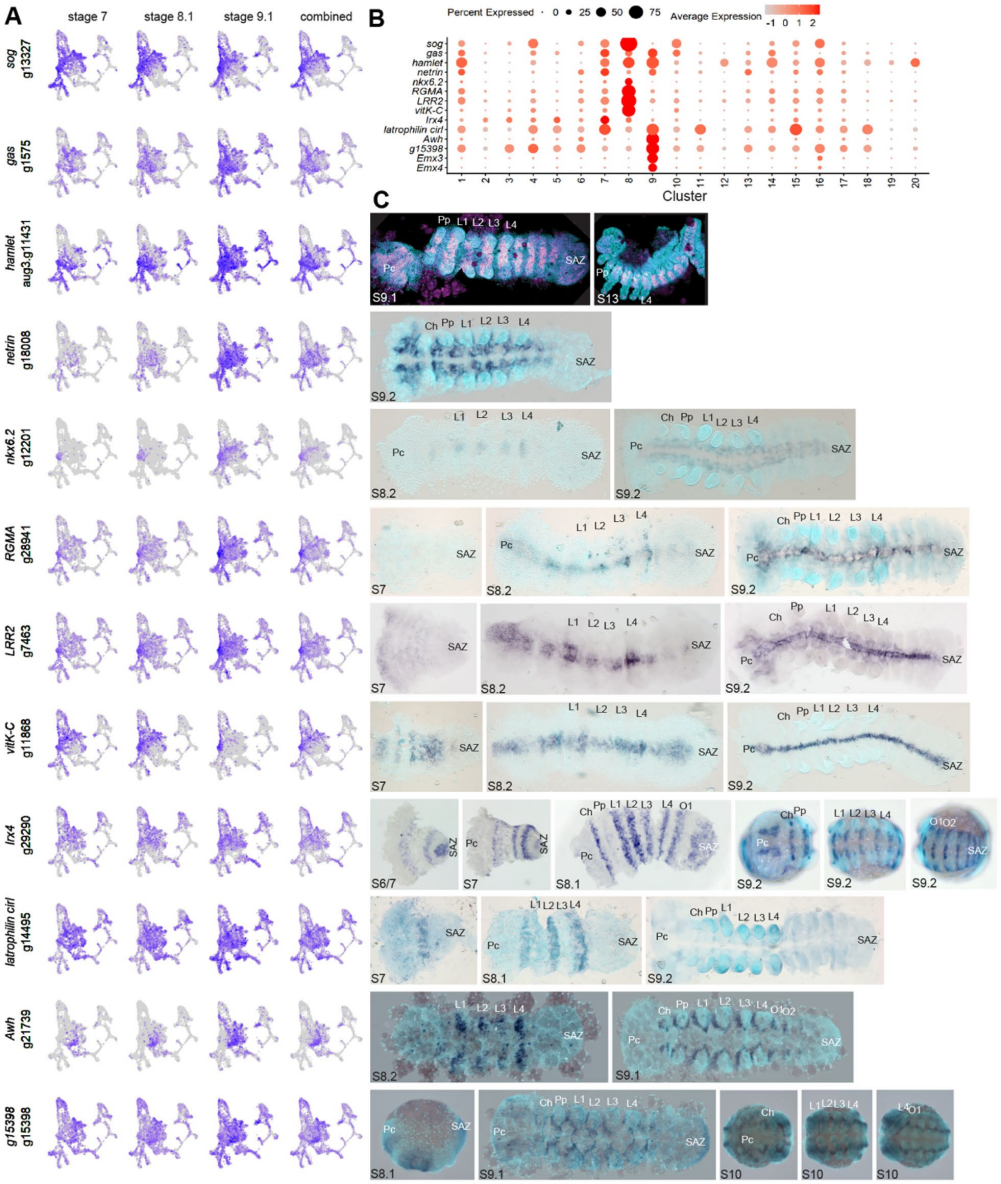
CNS and PNS-related clusters in P. tepidariorum. **A** UMAPs of each gene showing expression in rPCA integrated data, for each stage and combined. **B** Dotplot for markers of clusters that represent CNS and PNS cells. **C** RNA in situ hybridisations of marker genes for clusters 7, 8 and 9. Cluster 8 markers *Nk6.2*, *RGMA*, *LRR2 vitK-C* and *ham* are all expressed at the ventral midline. Clusters 7 and 9 markers show metameric patterns of expression around the ventral midline and appendages, which relate to putative PNS cells. *Pc* pre-cheliceral region, *Ch* cheliceral, *Pp* pedipalpal, *L1 to L4* leg-bearing 1 to 4, *O1 and O2* opisthosomal segments, *SAZ* segment addition zone

Following D-V axis formation during stages 5 and 6 [8, 12], patterning of the ventral midline during stages 7 to 9 is critical for the development of the nervous system. Therefore, we next explored our single cell data to gain deeper insights into this process. Ventral patterning in *P. tepidariorum* is regulated by expression of *sog* [*g13327*] [8]. The strongest expression and marker association (adjusted *p*-value) of *sog* is in cluster 8, although it was also a marker of clusters 4, 7, 10 and 16 (Fig. 5A, B). We, therefore, assayed the spatial expression of five additional cluster 8 marker genes (*Nkx6.2* [*g12201*], *RGMA* [*g28941*], *LRR2* [*g7463*], *vitK-C* [*g11868*] and *hamlet* (*ham*) [*aug3.g11431*]) (Fig. 5C). While the onset of their expression varied, they were all, like *sog*, expressed along the ventral midline and excluded from the posterior SAZ by stage 9 (Fig. 5A, B). Therefore, cluster 8 is related to the ventral midline. This suggests that while there are multiple *sog*-related ventral clusters, perhaps due to the broad expression of *sog* at stage 7 and 8.1 [8], cluster 8 was composed of cells that likely comprise the nerve cord cells, regulated by genes like *Nkx6.2*, which has a conserved role in specifying the ventral midline [66].

**Fig. 6:**
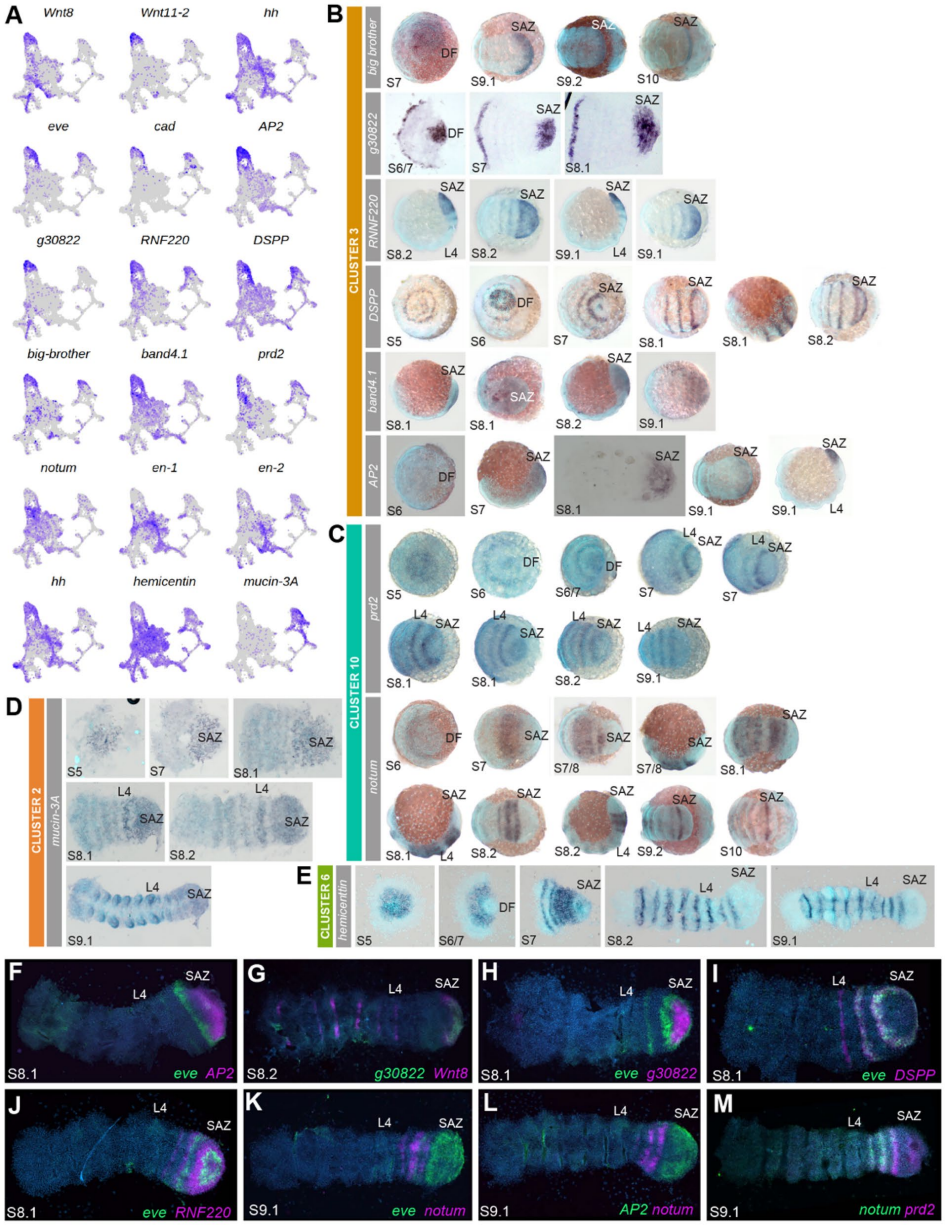
Multiple clusters relating to the SAZ and maturation of posterior segments. **A** UMAPs of markers and previously identified genes expressed in the SAZ. **B**–**E** Expression of markers for cluster 3 (**B**), cluster 10 (**C**), cluster 2 (**D**) and cluster 6 (**E**). **F**–**M** Double stainings, showing; **F**–**H** ‘static’ genes *Wnt8*, *AP2*, and *g30822*; (**I**) ‘dynamic’ gene *DSPP* in phase with *eve*, and (**J**) ‘dynamic’ gene *RNF220* out of phase with *eve*; **K**–**M** *notum* expression relative to *prd2* (**M**) to *eve* (**K**) and to *AP2* (**L**). *Pc* pre-cheliceral region, *Ch* cheliceral, *Pp* pedipalpal, *L1 to L4* leg-bearing segments 1 to 4, *SAZ* segment addition zone, *DF* dorsal field

Clusters 7 and 9 share the markers *ham*, *netrin* [*g18008*] (Fig. 5A, B and C and Fig. 6) and *growth arrested specification* (*gas*) [*g1575*] [51], which incidentally were also the only markers of cluster 1. All three of these genes had complex and broad expression across the embryo (Figs. 5A and 6C). Other cluster 7 markers *Irx4* [*g29290*] [47] and *latrophilin cirl* [*g14495*] were expressed at the anterior border in each segment. The cluster 9 markers, *Awh* [*g21739*], *g15398* (assayed here), *Emx3* [*g27623*] and *Emx4* [*g27624*] [47], all show metameric expression from the cheliceral to opisthosomal segments in ring-like patterns around the proximal base of each appendage at stage 9 (Fig. 5C). Cluster 9 is also marked by *Dfd-A* indicating that it may more specifically correspond to the prosomal leg-bearing segments (Fig. 3A). However, this may coincide with the earlier stage 8 expression of *Awh* and *g15398*, which are predominantly expressed in the prosoma.

The overlap between clusters 1, 7 and 9 may be due to issues regarding the broad distribution of cluster 1 cells that could disrupt marker prediction power. However, given the broad spatial expression of marker genes for clusters 7 and 9, these cells are likely also distributed across the embryo. Given the function of *ham* in the PNS, *Irx* genes in neural development [reviewed in 66] and *Awh* in (motor) neuron cell types, these clusters possibly relate to PNS cells that innervate appendages [68–71].

### New resolution of segment addition and maturation

A key process during stages 7 to 9 is the generation of most of the body segments. The pre-cheliceral, cheliceral and pedipalpal segments are instructed through expression wave-splitting, whereas leg-bearing segments of the prosoma are generated by the subdivision of the central region of the germband [51]. However, the twelve opisthosomal segments are added sequentially from the SAZ that develops from the posterior region of the germdisc and caudal lobe [25]. While candidate gene approaches and screens have provided insights into the regulation of opisthosomal segment generation from the SAZ [21, 25, 26], we explored our single cell data to try to better understand this further during stages 7 to 9.1.

We observed that posterior Hox genes have highest expression in clusters 3 and 10 (Fig. 3A). We then examined whether genes known to be expressed in the SAZ were also markers of these clusters. We found that *Wnt8* [*g19404*], *Wnt11-2* [aug3.*g1356*], *hh*, and *even-skipped* (*eve*) [*g21109*], were all markers of cluster 3, while *caudal* (*cad*) was also expressed in cluster 3 (Fig. 6A) [22, 25, 26, 25–26]. We also re-assessed the spatial expression of markers *AP2* [aug3.*g23531*] and *g30822* [aug3.*g27670*], which had been previously reported, and corroborated their expression in the SAZ (Fig. 6B) [51]. Furthermore, we analyzed the spatial expression of four additional markers of cluster 3 with previously unknown expression, *RNF220* [*g27156*], *dentin-like*/*DSPP* [*g3028*], *big-brother* [*g8835*] and *band4.1* [*g22446*] (Fig. 6B). Like *Wnt8*, *AP2* and *g30822* [26, 51], the expression of *big-brother* and *band4.1* was restricted to the posterior SAZ cells (Fig. 6B). In contrast, *RNF220* and *DSPP*, like *hh* and *eve* [22, 25], had dynamic expression in the SAZ and in stripes anteriorly in forming segments (Fig. 6B). Therefore, we were able to identify new genes from cluster 3 with posteriorly restricted expression that may maintain the SAZ as shown for *Wnt8* [26], and genes with cyclical expression associated with the sequential formation of new segments.

We analyzed the expression of two cluster 10 markers *paired2* (*prd2*) [*g10589*], and *notum* [*g17362*] (Fig. 6C) [74]. Note that *gsb*/*prd2* expression up to stage 8 was reported previously [51]. While these two genes were expressed in many regions of the embryo, they had similar expression in the opisthosomal region anterior to the SAZ, like both *Antp* genes at stage 9 (Fig. 6C) [14]. Additionally, *prd2* was also expressed in the posterior region of the SAZ (Fig. 6C).

To better understand the relationship and phasing of markers from these two clusters we assay the relative expression of marker genes from cluster 3 and 10. Some genes from cluster 3, like *Wnt8*, *AP2* and *g30822*, had overlapping expression restricted to the posterior of the SAZ, albeit with different anterior borders (Fig. 6F–H). Interestingly, we observed that some *AP2* and *g30822* expressing cells were mutually exclusive from *eve*, which suggests that cluster 3 includes cells that are no longer in the domain of *Wnt8*/*AP2*/*g30822* in the posterior SAZ but are in the striped domains more anterior (Fig. 6F–H). Genes like *eve*, *DSPP* and *RNF220* were all dynamically expressed, however, *DSPP* and *RNF220* exhibited different phasing relative to *eve* (Fig. 6I, J). This suggests that cluster 3 may represent cells across (a) whole segment(s), with different gene expression profiles prefiguring their sub-segmental A-P position (Fig. 6I, J). The expression of genes from cluster 10 overlapped (e.g., *notum* and *prd2*) but were mostly spatially distinct from cluster 3 genes (Fig. 6K–M), for example, neither *AP2* nor *eve* expression overlapped with *notum*. Therefore, cluster 10 likely represents cells of a transitioning zone where nascent segments have formed anterior to the SAZ and are maturing, which is consistent with *Antp* expression (Fig. 6K–M) [14].

*engrailed* (*en*) expression marks the parasegmental boundaries and posterior compartments of formed segments anterior to the SAZ [2, 22, 75, 76]. We found both *P. tepidariorum en* paralogs [*g24362* and *aug3.g15983*] were markers of cluster 6, which is also marked by *hh* and *hemicentin* [*g1871*] (Fig. 6E). *hh* and *hemicentin* are also expressed in the posterior compartment of all segments (Fig. 6E) [22, 51]. This suggests that cluster 6 represents cells in segments later during segmentation than in cluster 3 and 10. However, they only represent a subset of cells in each segment, and likely also relate to both prosomal and opisthosomal cells.

During assessment of markers, we observed that several cluster 3 markers, including *eve*, *DSPP* and *RNF220*, were also cluster 2 markers or at least expressed in cluster 2 (Fig. 6A). Cluster 2 was also marked by genes such as *SoxD2* [*g19045*] known to be expressed in the mesoderm of opisthosomal segments [50], as well as *FGFR1* [13], *integrin-alpha-8* [39] and *ush*, which were markers of dorsal and mesodermal clusters shown previously (Fig. 2B). In addition, *twist* was expressed in cluster 2 cells, indicating that at least some of these cells are mesodermal (Fig. 2C) [16]. We analyzed another cluster 2 marker, *mucin-3A* [*aug3.g13175*] and found that it was also expressed in the SAZ region and more anteriorly (Fig. 6D). This suggests that cluster 2 may represent cells that are internalised posteriorly and contribute to the mesoderm as segments are added [16, 17, 26].

Collectively, our data suggest cell clusters 2, 3, 6 and 10 are associated with posterior segmentation. These clusters relate to the SAZ and other cells more anteriorly that represent differentiating cells in formed segments. Furthermore, the cross-over between SAZ and mesodermal markers is consistent with the generation of multiple cell layers during posterior segmentation.

## Discussion

### A combination of ACME and SPLiT-seq for scRNA-seq developmental analysis

This study reports for the first time that a combination of ACME dissociation [29] and SPLiT-seq [41] is a very successful approach to obtain robust single cell data from arthropod embryos. ACME dissociation uses fixation of samples early in the dissociation process allowing us to collect multiple stages and samples that could be stored prior to single cell transcriptomics. This was highly desirable since embryogenesis progresses quickly in many arthropods and capturing distinct stages using lengthy (~ hours) dissociation processes might alter the single-cell expression. Furthermore, unfixed dissociated embryonic cells acquire stress altering their gene expression. Fixing embryos and cells with ACME stopped biological activity, therefore overcoming these hurdles, making the approach highly suited to study embryogenesis. We combined ACME with a variation of SPLiT-seq to obtain tens of thousands of single-cell transcriptomic profiles from multiple embryonic stages. This enabled us to capture new information about the genetic regulation and specification of the A-P and D-V axes, head patterning, mesodermal and endodermal lineages, the CNS and PNS, and posterior segmentation. Therefore, the combined application of ACME and SPLiT-seq is a powerful method to robustly profile early development.

### A single-cell atlas of spider development

Our scRNA-seq analysis of the spider *P. tepidariorum* has enabled a new understanding of spider embryogenesis at cellular resolution, including previously uncharacterized genes and unknown aspects of development. We used the integration of three developmental stages to define cell clusters that were represented by cells from all stages (Fig. 1). The assessment of markers between each stage and integrated data suggested that this approach did not drastically mask stage-specific cell clusters (Additional file 3: Figs. S5–S8), implying that between stage 7 and 9.1 no completely distinct cell states emerge, aside from the putative hemocyte cluster. From the integrated data, we defined 20 clusters, which represented known as well as novel cell states in multiple aspects of spider embryogenesis. We found multiple previously characterised genes in the clusters, which allowed us to establish their identities. Most cell clusters were also marked by genes that had not been previously studied and, therefore, offer new insights into these cells and the regulation of developmental processes. Collectively, our dataset constitutes the first cell state atlas through spider embryonic stages 7 to 9.1.

We detected fundamental aspects of developmental regulation relating to Hox patterning of the A-P axis (Fig. 3), the formation of the D-V axis (Figs. 2 and 5) and germ layer cell types (Fig. 2). These aspects reveal that the genetic components that regulate these core geometries also regionalise/cluster cells in our scRNA-seq data. The Hox genes were clearly segregated into three Hox positive groups, relating to pedipalpal, leg-bearing and opisthosomal segments (Fig. 3). Yet within each of these regions, ohnologs appeared to show divergence that might relate to sub- and/or neo-functionalisation, indicating a potential for scRNA-seq to study general trends of ohnolog evolution after WGD (Fig. 3 and Additional file 3: Fig. S9). The mesodermal/endodermal clusters carried a signature of increased G1 proportion, which suggests less proliferation compared to ectodermal clusters (Fig. 2). This potentially reflects the early determination of these cell types in spider development as shown in single cell data from earlier in embryogenesis [39] and that they are transcriptional very distinct at stages 7 to 9.1.

### Extra-embryonic cells not only contribute to the gut but also to potential hemocytes

The germdisc and germband of spiders has been largely considered to be exclusive from the extra-embryonic region and yolk. However, gut genes *serpent* and *hnf-4* [49], and the endodermal marker *GPCPD* [12], are expressed in the extra-embryonic region, suggesting these cells contribute to the spider endoderm. These genes were present in cluster 20, along with previously unknown markers that were also expressed in the extra-embryonic region (Fig. 2). By cell cycle gene scoring of clusters, we revealed that these cluster 20 cells are likely dividing less than many germband-related cell clusters.

While these endodermal cells related to cluster 20 have been previously reported, the expression of markers in the extra-embryonic region from cluster 19 suggests a need for reinterpretation of this tissue beyond only contributing to the gut. Prior to this study, the mesodermal heart gene *tinman* had been investigated in the spider *Cupiennius salei* [77], however, nothing was known about the genetic specification of peripheral circulatory cells in spiders. We found that cluster 19 expressed *Mef2.1*, which was later expressed in the heart (Fig. 2), as well as *hemocyanins.* This strongly suggests that cluster 19 may relate to heart, hemolymph and hemocyte cells. These cells initially surround the dorsal precheliceral region, but subsequently migrate into the extra-embryonic region. Interestingly, in *D. melanogaster*, embryonic hemocytes also originate from head mesoderm suggesting a perhaps conserved origin between chelicerates and insects [58–61]. In *D. melanogaster* and *Tribolium castaneum*, the extra-embryonic tissue is involved in immune response, and our identification of hematopoiesis markers suggest for the first time that cells in the extra-embryonic region of spider embryos could also contribute to this function [78, 79]. Thus, our data offer the opportunity to investigate blood/immune system evolution and function.

### Strong signal of D-V patterning in *P. tepidariorum* scRNA-seq

Like other arthropods, *P. tepidariorum* D-V axis formation and patterning is regulated by ventral *sog* and dorsal *dpp* signals [8, 9, 80]. In addition, genes like *Ets4*, *hh* and *fgf8* are also known to control cumulus migration, a process that is crucial for spider D-V axis formation [12, 13, 81]. However, there is still much to understand about the evolution of spider D-V patterning.

We observed a strong ventral signature of *sog* expression across cell clusters associated with the ventral germband (Fig. 5). As expected, *sog* and other cluster 8 markers, like *Nkx6.2*, were expressed along the ventral midline, as in other animals [66]. Clusters 7 and 9 were also marked by *sog* and other markers of these clusters exhibited complex expression likely associated with the PNS, given the function of genes like *ham*, *awh*, *Emx*, *Irx* and *netrin* [67–71]. Therefore, our single cell data revealed that alongside fundamental aspects of ventral specification, stages 7 to 9.1 also include patterning of the ventral PNS.

With respect to dorsal specification and patterning, genes expressed around the dorsal rim of the germband marked cluster 5 along with *dpp* (Fig. 2). Furthermore, there were differences between cell cycle scoring between these dorsal and ventral clusters, implying dorsal cells were not proliferating as much as ventral cells (Fig. 2). Curiously, we observed *noggin-D* expression in the cumulus, dorsal field, and dorsal region of the germ band (Fig. 2). The *Xenopus leavis noggin* homolog is expressed dorsally with *chordin*/*sog* [82] and ectopic expression can ventralise *D. melanogaster* embryos, thus revealing its conserved function as a BMP signalling inhibitor [82]. It is, therefore, surprising that *P. tepidariorum* exhibits dorsal expression of *noggin-D* where *dpp* signalling is active and necessary for dorsalisation [8]. This suggests that while *sog* and *dpp* play conserved roles in D-V specification in *P. tepidariorum,* other genes like *noggin-D* have diverged in function, indicating potential developmental system drift of D-V regulation in *P. tepidariorum*.

### New insights into pre-cheliceral region patterning

During prosomal development, *otd-1* helps specify the pre-cheliceral region [46], where structures/organs such as the brain, eyes and stomodeum develop. Markers of clusters 13, 14 and 16 showed specific spatial expression suggestive of demarcation of different pre-cheliceral regions (Fig. 4). For example, expression of *six3* paralogs in cluster 14 remained at the anterior rim and suggests conserved roles for these genes in forebrain development [65]. Furthermore, in several insects and the centipede *Strigamia maritima*, the combination of *six3* and *vsx* denotes the region of the anterior medial region that forms the *pars intercerebralis* [83–86]. Since *P. tepidariorum six3* paralogs and *vsx* are expressed in cluster 14 and anterior of the pre-cheliceral region, there is a possibility that *P. tepidariorum* shares a conserved regulatory control of *pars intercerebralis*. However, while neurogenic clusters were identified we were unable to definitively relate clusters to the non-neurogenic ectoderm that also forms at the anterior and lateral rim of the pre-cheliceral region [18, 19]. In the future, identification of these cells could give further insights into differentiation of the non-neurogenic ectoderm and eye development.

Expression of clusters 13 and 16 markers was observed initially at the anterior but subsequently shifted posteriorly to different extents and along the D-V axis (Fig. 4). Markers of these clusters, *lim1a*, *Pax6.1* and *Pax6.2*, are known for their essential roles in head and neural development in other arthropods and other animals [87–89]. Overall, our scRNA-seq reveals three cell clusters present from stage 7 that likely prefigure different regions of the head and brain (Fig. 4).

### Regionalisation of posterior segmentation based on genetic signatures of segment formation and maturation

Although previous studies identified key genes and their interactions regulating posterior segment addition in spiders and other arthropods [17, 25, 26, 25–26], we still have a poor understanding of how SAZs work. There is evidence that SAZs are likely genetically sub-structured, representing different regions and/or states that cells must progress through to form new segments [25, 97]. Our approach has identified more robust genetic signatures of SAZ sub-structure and revealed new genes involved in segmentation. This allows us to propose an extended model for the structure of the SAZ and segment addition.

Our data suggest that opisthosomal segmentation can be divided into four regions of segment formation (Fig. 6): region one relates to the most posterior cells in the SAZ, marked by genes like *Wnt8, AP2* and g30822 that have static expression [51]. These cells and several of the markers including *Wnt8*, can already be observed in stage 5 single cell data consistent with the SAZ forming from the caudal lobe during stages 5 and 6 [39]. As previously suggested, this posterior region of the SAZ probably represents a pool of undifferentiated cells that continuously contributes to new segments, perhaps analogous to the caudal region of the vertebrate presomitic mesoderm [26, 98].

Anteriorly in the SAZ, region two is marked by phased expression of pair-rule gene orthologs, like *eve* and *runt* [25], and newly identified genes including *RNF220* and *DSPP* (Fig. 6). These genes don’t appear to mark cell clusters at stage 5, which is consistent with these cell states only developing as the SAZ starts to generate segments during stages 6 and 7 [39]. These phased domains appear to broadly relate to A–P regions within forming segments (Fig. 6) [22, 25, 44, 51]. Interestingly, *RNF220* enhances canonical Wnt signalling in other animals and, therefore, might modulate the *Wnt8* activity in the SAZ of *P. tepidariorum* [99]. Region two, therefore, likely represents forming segments anteriorly from the SAZ, but still lacking expression of the segment polarity genes like *en* (Fig. 6) [2].

Region three is marked by expression of *notum,* which is also expressed in a similar pattern in *C. salei* [100]. *notum* is expressed in the posterior region of segments in this region (Fig. 6) and as it is a Wnt suppressor this gene may act on Wnt activity to facilitate segment maturation [26, 74]. Due to this transition from the SAZ to a more differentiated region we describe this region as the segment maturation zone (SMZ) (Fig. 6). Additionally, the expression of *sog* in cluster 10 also suggests that segmental patterning along the D-V axis is regulated anterior to the SAZ.

Formed segments express segment polarity genes like *en*, which marks the fourth region (Fig. 6) [2]. Indeed our data identified a clear genetic signature of cells in the posterior compartment of segments [76, 101, 102]. This includes expression of *hemicentin,* which is also expressed in developing somites of zebrafish [103], and knockdowns exhibit detachment phenotypes between cell types. Therefore, maturation of spider posterior segments may also involve Hemicentin mediated inter-cellular interactions between cells of multiple clusters defined in this study.

The SAZ cluster 3 is marked by several genes also expressed in cluster 2 (Fig. 6). Cluster 2 corresponds to mesodermal cells, given the expression of *SoxD2*, *FGFR1* and *integrin-alpha-8*, which are also mesodermally expressed in other animals [50, 104–106]. Both *integrin-alpha-8* and *SoxD2* are expressed in the SAZ and anterior to it, but also later are expressed in mesodermal metameric blocks in the opisthosoma and prosoma [50]. This is reminiscent of the *SoxD2* vertebrate homolog, *Sox5*, which is expressed in the presomitic mesoderm and later within each formed somite [104]. Furthermore, disruption of the SAZ by RNAi against *Wnt8* or *Dl* results in ectopic expression of the mesoderm gene *twist* in the SAZ [16, 17, 26]. This strongly suggests that there is dynamic specification and sorting of ectodermal and mesodermal cells via cell movement in the SAZ [105, 106]. Another marker of cluster 2, *Ubx-A*, has been shown to suppress *twist* in somatic myogenesis, which raises the possibility that this Hox gene may regulate ectoderm–mesoderm dynamics in the spider SAZ [107].

## Conclusion

Our scRNA-seq cell atlas of spider development corroborates previous findings and also provides novel insights into several important processes during spider embryogenesis. Future work to compare spider cell atlases to those of other chelicerates and other arthropods will also provide new insights into the evolution of cell differentiation and fate as well as the regulation of embryogenesis more broadly.

## Materials and methods

### Dissociation of *P. tepidariorum* embryos for single-cell sequencing

*P. tepidariorum* embryos were collected from egg sacs made by females from an inbred culture, staged [6] and dissociations performed as previously described [29]. Stage 7 (51–55 h after egglaying), 8.1 (56–55 h after egglaying) or 9.1 (76–80 h after egglaying) embryos were selected and weighed without silk to determine the sample size. Embryos were dechorionated with bleach (sodium hypochlorite, 5% active chlorine, Arcos) and tap water (1:1) then washed several times with ultrapure water (UltraPure^™^ DNase/RNase-Free Distilled Water, Invitrogen) to remove bleach traces. Unfertilized embryos were removed, and embryos were immersed in 10 ml ACME solution (3:1:2:14 of methanol, glacial acetic acid, glycerol, and ultrapure water). To break open the vitelline membranes and allow complete dissociation, embryos were treated with a few pulses of polytron homogenisation. Embryos in ACME solution were incubated for 1 h at room temperature (RT) on a rocking platform (Stuart SSL4) at 70 oscillations per minute. The cell suspension was filtered through a 50 μm filter (Sysmex/Partec CellTrics, Wolflabs) to separate remaining cell clumps and debris (e.g., vitelline membranes). Dissociated cells were pelleted at 1500 rpm for 5 min at 4 ℃. The supernatant was discarded, and the cell pellet washed with 7 ml PBS/1% Bovine Serum Albumin (BSA) (BSA Microbiological Grade Powder, Fisher BioReagents). The cells were pelleted at 1500 rpm for 5 min at 4 ℃, the supernatant was discarded, and the cell pellet resuspended in 1 ml 1 × PBS-1% BSA and DMSO (9:1) and stored at – 20 ℃.

### Flow cytometry and cell dilution

ACME-dissociated cells from stage 7, 8.1 and 9.1 embryos were thawed on ice. Thawed cells were centrifuged twice at 1500 rpm for 5 min at 4 ºC, to remove the DMSO, and resuspended in 400 μl of fresh 1 × PBS-1% BSA. Samples were then filtered through a 50 μm filter (Sysmex/Partec CellTrics, Wolflabs) and collected into new 1.5 ml Eppendorf tubes, on ice. 50 μl of filtered cells were added to 100 μl of 1 × PBS-1% BSA. The remaining undiluted samples were kept on ice, in the fridge, for the rest of the analysis.

Dilutions were stained with 0.4 μl of DRAQ5 (5 mM stock solution, Bioscience) and 0.8 μl of Concanavalin-A conjugated with AlexaFluor 488 (1 mg/ml stock solution, Invitrogen), and incubated in the dark for 25 min at room temperature. DRAQ5 was used as nuclear dye, while Concanavalin-A (Con-A) was used as cytoplasmic dye. We visualized and counted cells using a CytoFlex S Flow Cytometer (Beckman Coulter). For each stained dilution, we made three measurements of 10 μl and registered the average number of total events (total ungated population). From this, we calculated the number of total events per μl in our undiluted samples.

When multiple samples were available, we selected those with the highest percentage of singlets (DRAQ5-positive & Concanavalin-A-positive single-cells). To obtain this percentage of singlets, we used the following gating strategy: FSC-H vs FSC-A, where we selected only well-correlated events (first filter to remove aggregates); Con-A vs FSC-A, where we selected Con-A positive events (events with cytoplasm); DRAQ5 vs FSC-A, where we selected DRAQ5 positive events (events with nucleus); DRAQ5-A vs DRAQ5-H, where we selected only well-correlated events (second filter to remove aggregates); and DRAQ5 vs Con-A, where we obtained the final number of singlets.

To prepare the cells for the SPLiT-seq protocol, samples were diluted in fresh 0.5 × PBS buffer to a final concentration of 625 events/μl and kept on ice.

### Re-annotation of *P. tepidariorum* genome for mapping SPLiT-seq data

SPLiT-seq has a bias towards capturing the 3ʹ region of transcripts. To ensure capture of the signal from the SPLiT-seq data we re-annotated the genome (GCA000365465.2) of *P. tepidariorum*. Bulk RNA-seq data [108] were combined with multiple paired end libraries from a range of other embryonic stages. All data were quality trimmed with Trimmomatic v0.39 [109] and then mapped to the genome using Star v2.7.9a [110] using the 2-pass method for better detection of splice junctions. Alignment information was used as evidence for Braker v2 [111] annotation, with ten rounds of optimisation, UTR training and considering CRF models. This annotation was combined with the previous genome annotation [14] by first merging the gene coordinates with bamtools merge. Gene models with a new annotation were replaced. Those with multiple new annotations to one previous annotation were rejected and the previous annotation was retained. New annotations that compounded multiple previous annotations were retained. This final annotation contained 33,413 gene models compared to the 27,950 in the Schwager et al*.* (2017) annotation. The majority (18,544) of these genes show a 1:1 relationship between annotations, as well as additional annotations captured by the new version, and the fusion of split genes by merging annotations. Old annotations are given as aug3.g* whereas new annotations are given as g*. The reannotation GTF and amino acid fasta files have been uploaded to figshare (https://doi.org/10.6084/m9.figshare.c.6032888.v2). Gene orthologues were estimated by alignment scoring using Diamond tblastx [112, 113] to the NCBI nr database.

### Mitochondrial genome assembly of *P. tepidariorum*

Mitochondrial expression in single-cell sequence data can be indicative of cell stress and therefore a useful metric to measure. We assembled a near complete version of the mitochondrial genome to be included in the mapping steps. DNA-seq data (SRR891587) from the genome assembly was trimmed with Trimmomatic v0.39 [109] and assembled with Spades v3.13.1 [114] using kmer sizes 21, 33, 55, 77 with the *P. tepidariorum* CO1 sequence (DQ029215.1) as a trusted contig. The spades contig matching the CO1 sequence was extracted and then extended with NOVOPlasty v4.2 [115] to achieve a final assembly of length 14,427 bp, though it was not circularisable. MiToS v2 [116] identified all expected features. The sequence was added to the genome assembly and a feature spanning the full length was added to the GTF gene coordinates file for mapping. The mitochondrial genome assembly has been uploaded to figshare (https://doi.org/10.6084/m9.figshare.c.6032888.v2).

### SPLiT-seq, filtering, pre-processing, and clustering analysis

The SPLiT-seq protocol was performed as previously described with some modifications (Additional file 1) [29]. Libraries were sequenced with 150 bp paired-end Illumina NovaSeq 6000 S4 flow cell, provided commercially by Novogene. Raw reads have been uploaded to the ENA with BioProject PRJEB53350.

Total sequencing output was 103.9 + 40.7 Gb, constituting a total of 963,482,454 raw reads, with > 99.98% clean reads and a Q20 > 93.94%. All data and samples passed FastQC inspection. Adapters and low-quality bases were trimmed with Cutadapt v1.18 [117] and properly paired reads were combined with Picard FastqToSam v2.20.5. All sequence runs were combined with Picard MergeSamFiles to attain paired reads for downstream expression analysis. To generate reference files, first Picard CreateSequenceDictionary was used to generate a dictionary from the genome plus mitochondrial sequence and re-annotations. Then converted to a RefFlat, a reduced GTF and intervals with DropSeq v2.4.0 [118] tools ConvertToRefFlat, ReduceGtf and CreateIntervalsFiles, respectively. For mapping the data, a Star v2.7.9a [110] genome index was generated with sjdbOverhang 99. These reference files and genome index were used as inputs for the Split-seq_pipeline.sh [118]. An expression matrix was generated with dropseq DigitalExpression, including reads mapping to introns, with a barcode edit distance of one, and outputting cells that had at least 100 genes. Cells from each stage were extracted from this matrix using the 16 sequences from cell barcode one.

Each library per stage was first processed for doublet removal. The expression matrix for each stage was loaded into Seurat v4 [119] and subset to contain cells where genes are expressed in at least 20 cells; that have genes numbers between 400 and 1800; UMI counts between minimums of 650 for stage 7, 700 for stage 8.1 and 500 for stage 9.1 and maximum of 4500; and no more than 1% mitochondrial expression. This initial dataset contained cells for stages 7 (1967 and 3111), 8.1 (2058 and 3029) and 9.1 (3866 and 5459), for libraries one and two, respectively. Each sample was normalized with SCTransform with the glmGamPoi method and variable features threshold of 1.3 and regressing the mitochondrial expression, UMI counts and gene counts. 50 PCs and neighbours were computed using k. param 100, and clusters were identified at a resolution of 1 for stage 7 and 8.1 and 1.2 for stage 9.1. Using doubletFinder v3 [120], 5% doublets we removed, identifying an appropriate pK with an initial parameter sweep, and retained singlets were extracted.

Doublet filtered stage specific matrices were then processed for integration in Seurat, normalising with SCTransform using the “glmGamPoi” method and a variable feature threshold of 1.3 and regressing the mitochondrial expression, UMI counts and gene counts. All samples were integrated using the reciprocal PCA method (50 PCs) and 40 anchors. 50 PCs were computed for the integrated data and used for UMAPs with the “umap-learn” method and 100 n.neighbors, 0.3 min.dist, 42 seed and the “correlation” metric. Nearest neighbours were determined using 100 k.param and 50 n.trees. The Leiden algorithm was used for clustering with a resolution of 1.2. The clustering resolutions were guided by ChooseR [121] and clustree [122] analysis. FindAllMarkers was used to extract markers for each cluster using the Wilcoxon method and including genes that were expressed in at least 25% of the cells in their respective cluster and a return threshold of 1e-5. Marker genes were annotated initially with the NCBI nr database using Diamond v2.0.8.146 [112, 113] and refined for existing genes already characterised in *P. tepidariorum*. Hierarchical grouping of clusters was performed using BuildClusterTree from Seurat and ggtree [123] to visualise. Seurat objects available upon request.

### Clustering and marker comparisons

Clustering from different Seurat processing iterations was compared using the adjustRandIndex from the R package mclust [53]. Raw count matrices were processed similarly with a variable gene threshold, ranging from 1.2 to 1.7, and the integration k.anchor ranging from 5 to 45. Cluster IDs were extracted and for an all versus all comparison of clustering similarity scored between 0 and 1 and were visualized with pheatmap in R. UMAP coordinates were extracted and plotted with ggplots in R.

To compare marker lists of clusters from different runs, FindAllMarker was used with 25 percent expressed in cluster and 1e-5 *p*-value threshold parameters and compared using a hypergeometric distribution test. The total number of genes in the spider single cell expression matrix was used as the pool from which genes could be selected. The markers and overlap were computed from each cluster, and these values were used in a sum(dhyper()) R function with a Bonferroni adjusted *p*-value. Code for the hypergeometric distribution test can be found at https://github.com/djleite/Hypergeo_SingleCell_Markers. We also used ClusterMap to compare the relationship between clusters from different datasets [54].

### Cell cycle gene scoring

The cell cycle gene scores of *P. tepidariorum* were estimated in Seurat. To identify cell cycle genes the G2/M and S phase *D. melanogaster* gene IDs were obtained from (https://github.com/hbc/tinyatlas/blob/master/cell_cycle/Drosophila_melanogaster.csv) and extracted from the release v6.49 of the *D. melanogaster* proteins from FlyBase. Spider orthologs were identified using a default Diamond blastp [112, 113] search, selecting the two best hits. Spider cell cycle gene IDs were used with Seurat CellCycleScoring to identify cell cycle phasing. Fasta sequence of *P. tepidariorum* cell cycle genes is provided in Additional file 2.

### Gene cloning and expression analysis

For gene expression characterization in *P. tepidariorum* embryos, we performed colorimetric in situ hybridisation (ISH) [124], fastred [20] and double fluorescent in situ hybridisation (dFISH) [50] as previously described with minor modifications (Additional file 3).

### Supplementary Information

**Additional file 1. **Marker genes list for each stage 7, 8.1 and 9.1, and for non-integrated and rPCA integrated clusterings.

**Additional file 2. **FASTA file of predicted cell cycle genes in *Parasteatoda tepidariorum*.

**Additional file 3. **Supplementary Text, Tables and Figures.

## Data Availability

Raw reads for the scRNA-seq have been uploaded to the ENA with BioProject PRJEB53350. The re-annotation of the genome, the mitochondrial genome assembly of *P. tepidariorum* and the scRNA-seq expression matrices have been uploaded to figshare (https://doi.org/10.6084/m9.figshare.c.6032888.v2). Additionally, resources and scripts to plot markers are in figshare (https://doi.org/10.6084/m9.figshare.24899643.v1).
